# Associations between body mass index and all-cause and CVD mortality in agriculture, forestry, and fishing occupations: A prospective cohort study using NHANES data (1999–2014)

**DOI:** 10.1371/journal.pone.0305922

**Published:** 2024-07-08

**Authors:** Yanmeng Qi, Baoshan Zhang, Han Yang

**Affiliations:** 1 International Medical Department, Xidan Campus, Peking Union Medical College Hospital, Chinese Academy of Medical Sciences and Peking Union Medical College, Beijing, China; 2 College of Environmental Science and Engineering, China West Normal University, Nanchong, China; 3 Key Laboratory of Sustainable Forest Management and Environmental Microorganism Engineering of Heilongjiang Province, Northeast Forestry University, Harbin, China; Instituto Nacional de Cardiologia Ignacio Chavez, MEXICO

## Abstract

**Introduction:**

Obesity, as indicated by elevated Body Mass Index (BMI), is a well-established global health concern associated with increased morbidity and mortality across diverse populations. However, the influence of BMI on individuals in Agriculture, Forestry, and Fishing (AFF) occupations, characterized by unique challenges and environmental factors, has received limited research attention.

**Methods:**

Our study, a prospective cohort analysis, utilized National Health and Nutrition Examination Survey (NHANES) data from 1999–2014, targeting adults above 18 in AFF occupations with comprehensive BMI data, omitting individuals with a history of cancer. Mortality outcomes were extracted from the NHANES mortality file, and BMI was segmented into eight categories. Essential covariates such as age, sex, race, and various health factors were incorporated. The statistical analysis encompassed Cox regression, generalized additive models, smooth curve fitting, and stratified analyses.

**Results:**

During 1,005 person-years with 201 all-cause and 57 CVD deaths, we observed L-shaped and U-shaped correlations of BMI with all-cause and CVD mortality, featuring a pivotal inflection at 26.69 and 27.40 kg/m^2^. Above this BMI threshold of 26.69 and 27.4 kg/m^2^, all-cause mortality association was not significant while CVD mortality was positive.

**Conclusions:**

This study highlights a unique BMI-mortality association in AFF occupations, diverging from standard patterns. The rigorous labor and environmental conditions in AFF jobs suggest that a certain range of higher BMI could reduce mortality risk. This highlights the necessity for tailored health guidelines in different occupations. Future research should concentrate on diverse health indicators and enhanced risk assessment for physically strenuous occupations.

## Introduction

Obesity, defined by the World Health Organization (WHO) in 1995 as having a Body Mass Index (BMI) of ≥ 30 kg/m^2^, is a globally recognized health concern characterized by elevated BMI and associated with increased morbidity and mortality across diverse populations [1–3]. The association between high BMI and adverse health outcomes, including all-cause mortality, has been extensively explored in epidemiological studies [4–6]. Nonetheless, the influence of BMI on individuals with particular occupational backgrounds, including those in Agriculture, Forestry, and Fishing (AFF) occupations, has received limited research attention. Data analysis from the 2004–2013 National Health Interview Survey revealed that the average Body Mass Index (BMI) of American workers stood at 27.6 kg/m^2^ [7]. Specifically, workers in agriculture, forestry, and fishing had an average BMI of 28.3 kg/m^2^, moderately high relative to other sectors. In the AFF sectors, BMI critically assesses worker mortality [8, 9]. Work in these industries often requires high-intensity physical labor, making extreme BMI levels—either high or low—harmful to an individual’s physical capacity and efficiency. Specifically, high BMI increases the risk of cardiovascular diseases [7], while low BMI can cause malnutrition and impaired immune function [9]. Excessive weight reduces flexibility and balance, increasing accident risks when operating heavy machinery or working on unstable terrains [10]. Workers in these sectors are at increased risk of chronic diseases, exacerbated by the unique demands of their work and living environments and closely linked to unhealthy BMI levels [11]. Therefore, monitoring and managing BMI in these workers is essential for improving their health and safety. This distinct group encounters specific challenges and environmental factors that may potentially alter the association between BMI and mortality [12].

Epidemiological studies have consistently shown that individuals with a high BMI face an increased risk of all-cause mortality compared to those with a normal BMI [13, 14]. The relationship between excess body weight and mortality is believed to be underpinned by various physiological and metabolic mechanisms [15, 16]. These include an increased likelihood of developing chronic conditions, such as type 2 diabetes, cardiovascular diseases, and certain cancers, which collectively contribute to the overall mortality burden [17]. Furthermore, the effects of obesity on immune function and inflammation may play a pivotal role in the increased susceptibility to infectious diseases, as exemplified during the COVID-19 pandemic [18]. Given the profound implications of obesity on public health, it is imperative to comprehensively investigate the BMI-mortality link in subpopulations characterized by unique lifestyles and occupational exposures.

Agriculture, Forestry, and Fishing (AFF) occupations represent a vital sector of the global workforce, providing essential resources that sustain human livelihoods [19]. The men and women employed in these industries are often subject to rigorous physical demands, prolonged exposure to environmental hazards, and irregular working hours [20]. The inherent factors specific to AFF occupations may significantly impact the health and well-being of these individuals [21]. We are particularly interested in examining the possible relationship between BMI and the health outcomes of individuals in AFF professions. The nature of AFF occupations frequently demands substantial physical exertion, which might influence a distinctive BMI-mortality association. Workers in these occupations may exhibit diverse BMI distributions influenced by the strenuous physical demands of their jobs, dietary habits, and other lifestyle factors. Additionally, exposure to environmental hazards, such as agricultural pesticides or extreme weather conditions in fishing, can introduce additional complexities to the BMI-mortality relationship [22, 23].

Currently, there is a lack of research specifically addressing the association of BMI with all-cause and CVD mortality in the AFF occupation cohort. The lack of information emphasizes the necessity for a thorough investigation into how this distinct occupational environment may influence the well-established BMI-mortality relationship. This prospective cohort study, using data from the 1999–2014 National Health and Nutrition Examination Survey (NHANES) database, seeks to clarify the association between BMI and all-cause and CVD mortality among individuals in the AFF industries. Through the analysis of this cohort, we aim to offer valuable insights into whether the BMI-mortality association among AFF workers differs from the general population, and the extent to which occupation-specific factors may influence this relationship.

## Methods

### Study design and participants

This prospective cohort study employed data sourced from the National Health and Nutrition Examination Survey (NHANES), conducted between 1999 and 2014 by the Centers for Disease Control and Prevention (CDC) and the National Center for Health Statistics (NCHS). NHANES is a nationally representative survey that collects data on the health and nutritional status of the U.S. civilian noninstitutionalized population using a complex, multistage probability sampling design. For this analysis, we focused on adults aged 18 years and older who self-reported their longest job as AFF. The NCHS Occupational Classification Source and Census Bureau Occupation Classification Source Codes were used to encode the occupation data from the NHANES for the periods 1999–2004 and 2005–2014, respectively.

**Fig 1:**
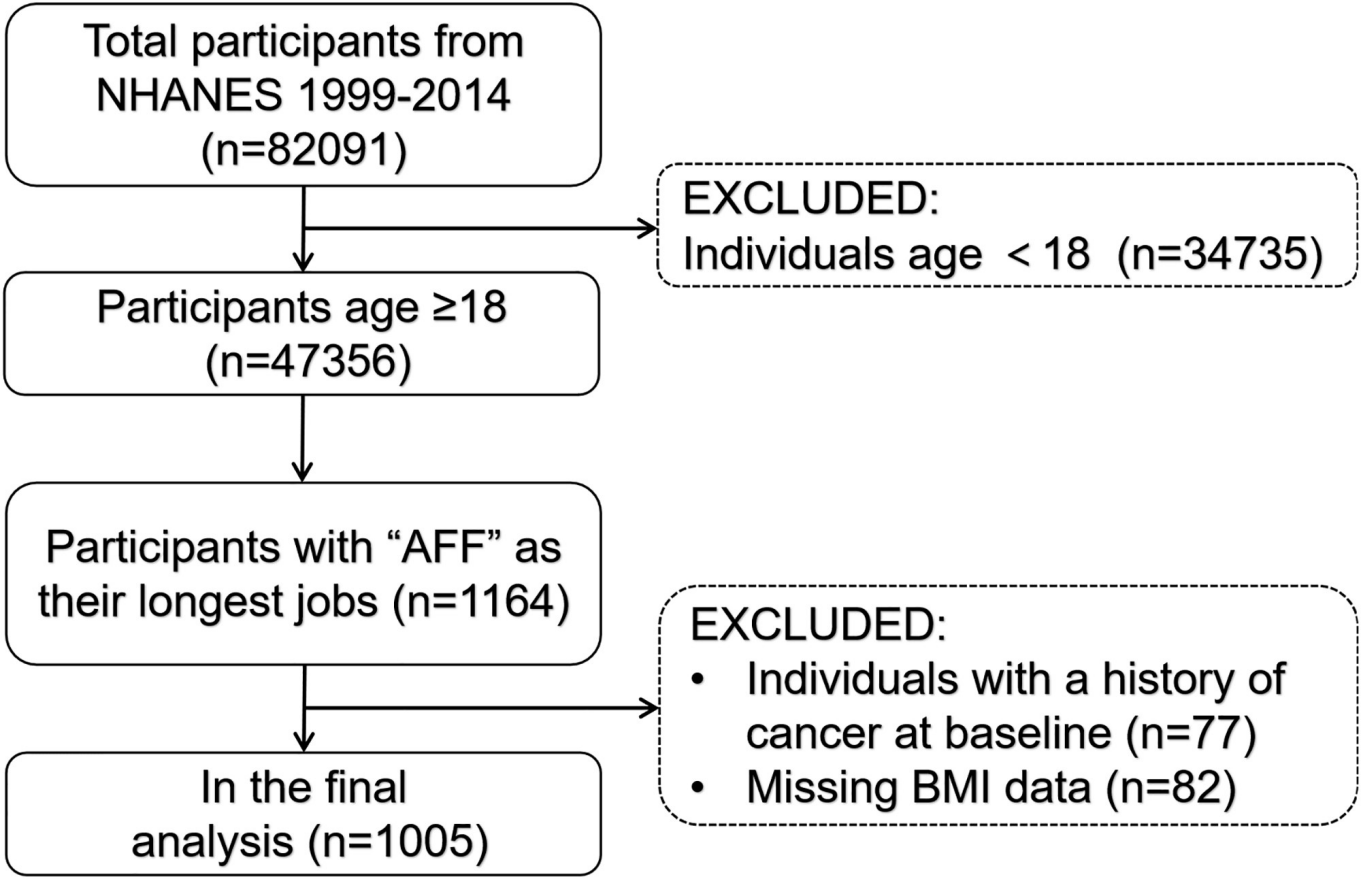
Flow chat of sample selection from the NHANES.

A total of 47356 participants aged 18 and older were recruited, from which we selected 1164 individuals with AFF as their longest jobs (Kind of work you have done the longest; Occupation group code: longest job). After excluding participants with a history of cancer at the baseline (Have you ever been told by a doctor or other health professional that you had cancer or a malignancy of any kind?) (n = 77) and incomplete BMI data (n = 82), our analysis included a final sample of 1005 participants with complete data. A schematic representation of the sample selection process is depicted in Fig 1. NHANES protocols were approved by the National Center for Health Statistics ethics review board, and written informed consent was obtained from all participants. This study followed the Strengthening the Reporting of Observational Studies in Epidemiology (STROBE) Statement guidelines. (https://www.equator-network.org/reporting-guidelines/strobe/).

The NHANES study obtained ethical clearance from the Research Ethics Review Board of the National Center for Health Statistics and the documented consent was obtained from participants (Protocol #98–12, #2005–06, #2011–17). Written informed consent was obtained from all participants. Further information can be found at https://www.cdc.gov/nchs/nhanes/irba98.htm.

### Outcome variable

The primary outcome variable for this study were all-cause and CVD mortality. Mortality outcomes were determined through the linkage of NHANES data with the National Death Index (NDI). The linked mortality file provides information on the date and cause of death for NHANES participants who have died. Participants were followed from the date of their NHANES interview until their date of death or the end of the study period (December 31, 2015), whichever came first. Cause-specific mortality was ascertained by the International Statistical Classification of Diseases and Related Health Problems, Tenth Revision (ICD-10). CVD mortality was classified as death caused by diseases of heart (ICD-10, codes 054–068).

### Exposure variable

The primary exposure variable in this study was BMI, which is calculated as an individual’s weight in kilograms divided by the square of their height in meters (kg/m^2^), both of which were obtained during the physical examination. Subsequently, based on previous research, the data was then categorized into eight groups for analysis in our study: <18.5, 18.5–20.4, 20.5–22.4, 22.5–24.9, 25–27.4, 27.5–29.9, 30–34.9, and ≥35 [24].

### Assessment of covariates

Sociodemographic and lifestyle variables were collected and categorized, including age, sex (males or females), race (Hispanic or non-Hispanic populations, including non-Hispanic Black, non-Hispanic White, and others), smoking status (whether smoked at least 100 cigarettes in life), heavy alcohol status (ever have 4/5 or more drinks every day), vigorous work activity (yes or no), and the presence of chronic diseases. Hypertension, diabetes and arthritis were self-reported by participants who had received a diagnosis from a health professional (Ever told by a doctor that you have hypertension/diabetes/arthritis). Metabolic indicators and laboratory variables were obtained from the NHANES laboratory component, following established protocols accessible on the NHANES website.

### Statistical analyses

The data in this study were statistically analyzed in accordance with CDC guidelines [25]. Descriptive statistics were used to summarize the baseline characteristics of the study population, including mean and standard deviation (SD) for continuous variables and proportions for categorical variables. Cox proportional hazards regression models were employed to assess the association between BMI and mortality, adjusting for the aforementioned covariates. Model 1 was non-adjusted. Model 2 was adjusted for age, sex and race. Model 3 was adjusted for age, sex, race, arthritis, smoking status and heavy alcohol. Crude and adjusted hazard ratios (HRs) and their corresponding 95% confidence intervals (CIs) were estimated.

To explore potential non-linear associations between BMI and mortality, generalized additive model were employed. Smoothed curve fitting techniques were applied to visualize the relationship between BMI and all-cause and CVD mortality, allowing for the identification of critical inflection points or thresholds. Stratified analyses were conducted to examine whether the observed associations between BMI and all-cause mortality varied across subgroups defined by sex, age (<60 years old or ≥60 years old), race (Hispanic or non-Hispanic), hypertension, diabetes, arthritis, smoking status and heavy alcohol. And age, sex, race, arthritis, smoking status and heavy alcohol were all adjusted except the variable itself in stratified analyses.

All statistical analyses adhered to NHANES analytical guidelines and were conducted using the R statistical package (R 3.4.3) and Empower Stats. Statistical significance was established at p-values < 0.05.

## Results

### Baseline characteristics of study participants

The baseline characteristics of the study population, stratified by BMI category, are summarized in Table 1. Age increased with higher BMI categories after the first group. The proportion of males decreased and the representation of Hispanics increased with higher BMI categories. There was an observed increase in the prevalence of diabetes, hypertension, and arthritis across higher BMI groups. The highest rates of smoking and heavy alcohol consumption were in the BMI <18.5 kg/m^2^ group, but the proportion of smokers decreased and the proportion of heavy drinkers first increased and then decreased with higher BMI categories. For metabolic indicators, levels of Triglycerides (TG), Total Cholesterol (TC), LDL Cholesterol (LDL-C), arm circumference, waist circumference, uric acid, serum glucose, and glycohemoglobin increased with higher BMI categories, while HDL Cholesterol (HDL-C) levels decreased. However, the increase in LDL-C was not statistically significant across BMI groups. Also, there were no statistically significant differences in creatinine and blood urea nitrogen levels across BMI groups, although both peaked within the BMI range of 22.5–25.0 kg/m^2^.

**Table 1:** Baseline characteristics of participants with AFF according to BMI.

Characteristics	Body mass index (kg/m^2^)								P value
	<18.5	18.5–20.5	20.5–22.5	22.5–25.0	25.0–27.5	27.5–30.0	30.0–35.0	≥ 35.0	
N.	14	40	75	168	184	181	222	121	
Length of follow-up (months)	133.2 ± 66.8	169.2 ± 67.1	162.6 ± 70.1	165.5 ± 64.3	161.9 ± 55.8	154.5 ± 62.4	162.4 ± 62.9	152.1 ± 58.5	0.266
Age (years)	45.5 ± 18.6	36.3 ± 22.3	42.1 ± 22.6	44.8 ± 21.4	46.6 ± 18.9	47.6 ± 19.0	48.9 ± 18.9	49.3 ± 15.9	0.002
Triglycerides (mmol/L)	1.2 ± 0.8	1.0 ± 0.7	1.3 ± 0.9	1.6 ± 1.3	2.1 ± 1.9	1.9 ± 1.3	2.2 ± 1.4	2.0 ± 1.3	<0.001
Total Cholesterol (mmol/L)	4.7 ± 0.8	4.7 ± 1.2	4.8 ± 1.1	4.9 ± 1.1	5.1 ± 1.1	5.3 ± 1.1	5.2 ± 1.1	5.2 ± 1.1	<0.001
LDL Cholesterol (mmol/L)	2.4 ± 1.1	2.9 ± 1.0	2.9 ± 0.9	3.0 ± 1.0	3.0 ± 0.9	3.3 ± 0.8	3.1 ± 0.9	3.1 ± 0.9	0.163
HDL Cholesterol (mmol/L)	1.7 ± 0.3	1.4 ± 0.4	1.4 ± 0.4	1.3 ± 0.4	1.3 ± 0.3	1.2 ± 0.3	1.2 ± 0.3	1.2 ± 0.3	<0.001
Arm circumference (cm)	24.6 ± 1.7	26.9 ± 2.0	28.3 ± 2.0	29.7 ± 2.0	31.6 ± 1.9	33.3 ± 2.3	35.2 ± 2.5	40.0 ± 3.9	<0.001
Waist circumference (cm)	72.4 ± 5.4	76.4 ± 4.4	80.8 ± 6.2	87.0 ± 6.4	94.0 ± 6.2	100.5 ± 6.5	107.4 ± 7.0	122.3 ± 11.7	<0.001
Creatinine (μmol/L)	74.5 ± 17.6	78.8 ± 22.8	71.2 ± 16.5	86.7 ± 88.7	76.5 ± 21.2	77.5 ± 56.7	70.8 ± 19.9	72.8 ± 25.6	0.086
Blood urea nitrogen (mmol/L)	3.9 ± 1.9	4.9 ± 1.9	4.4 ± 1.8	5.2 ± 2.3	5.0 ± 2.0	5.0 ± 1.8	4.9 ± 1.8	5.0 ± 2.7	0.176
Serum uric acid (μmol/L)	295.6 ± 105.6	321.2 ± 95.1	288.5 ± 70.6	324.5 ± 80.2	320.6 ± 78.1	328.0 ± 75.8	335.4 ± 81.4	369.6 ± 93.5	<0.001
Serum glucose (mmol/L)	4.9 ± 0.4	4.8 ± 0.6	5.3 ± 2.3	5.6 ± 2.1	5.8 ± 2.4	5.4 ± 1.3	6.0 ± 2.4	6.1 ± 2.3	<0.001
Glycohemoglobin (%)	5.3 ± 0.3	5.3 ± 0.4	5.4 ± 1.1	5.6 ± 1.2	5.7 ± 1.2	5.6 ± 0.8	6.0 ± 1.4	6.0 ± 1.0	<0.001
Sex (N %)									<0.001
Males	12 (85.7%)	32 (80.0%)	55 (73.3%)	136 (81.0%)	150 (81.5%)	132 (72.9%)	141 (63.5%)	66 (54.5%)	
Females	2 (14.3%)	8 (20.0%)	20 (26.7%)	32 (19.0%)	34 (18.5%)	49 (27.1%)	81 (36.5%)	55 (45.5%)	
Race (N %)									<0.001
Hispanic	4 (28.6%)	19 (47.5%)	39 (52.0%)	100 (59.5%)	130 (70.7%)	119 (65.7%)	145 (65.3%)	64 (52.9%)	
Non-Hispanic	10 (71.4%)	21 (52.5%)	36 (48.0%)	68 (40.5%)	54 (29.3%)	62 (34.3%)	77 (34.7%)	57 (47.1%)	
Hypertension (N %)	3 (21.4%)	3 (8.1%)	14 (20.0%)	35 (22.4%)	40 (22.2%)	41 (22.9%)	77 (36.2%)	47 (39.2%)	<0.001
Diabetes (N %)	0 (0.0%)	3 (7.5%)	4 (5.3%)	15 (9.0%)	19 (10.3%)	23 (12.7%)	36 (16.3%)	24 (19.8%)	0.01
Arthritis (N %)	6 (50.0%)	4 (14.3%)	8 (13.1%)	27 (18.4%)	33 (19.2%)	39 (23.1%)	51 (24.9%)	39 (33.9%)	0.004
Smoking status (N %)	10 (83.3%)	17 (58.6%)	30 (49.2%)	68 (46.6%)	82 (48.0%)	84 (49.4%)	85 (41.3%)	42 (36.2%)	0.023
Heavy alcohol (N %)	6 (60.0%)	2 (10.0%)	13 (26.5%)	35 (31.8%)	35 (25.5%)	43 (31.9%)	32 (20.6%)	17 (20.5%)	0.020

**Notes**: Mean ± SD for continuous variables and N (%) for categorical variables. P values were calculated by Student T test or Mann-Whitney test, depending on the parametric or non-parametric distribution of continuous data.

### Relationships of BMI with mortality

**Fig 2:**
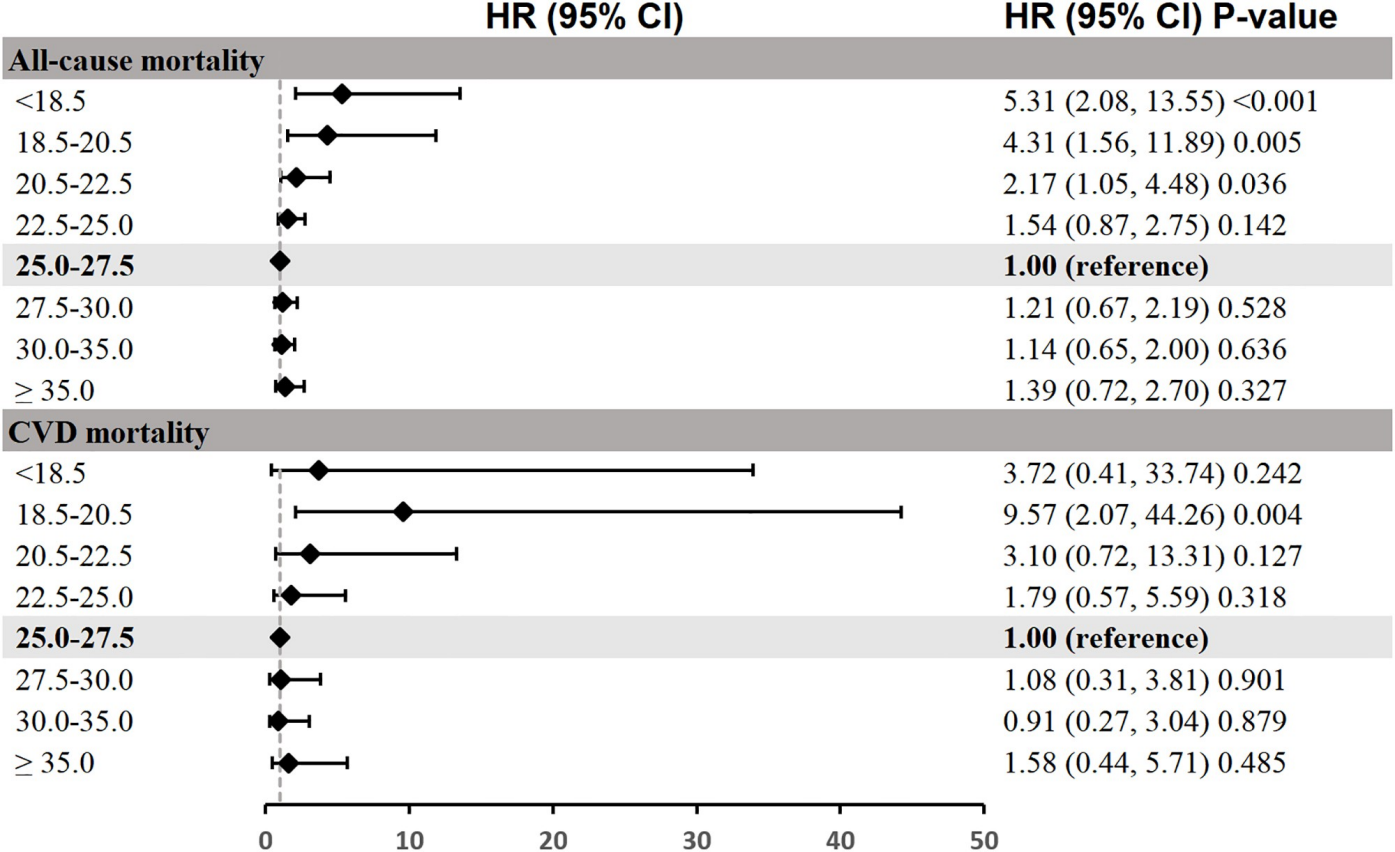
Forest plot depicting the association between BMI categories and mortality. Each point represents the HR for mortality within a specific BMI category, with horizontal lines denoting the 95% CIs. The reference category (BMI 25.0–27.5 kg/m^2^) is indicated by the diamond symbol. HRs are adjusted for age, sex, race, arthritis, smoking status, and heavy alcohol use as per Model 3 of the Cox regression analysis. A HR greater than 1 suggests an increased risk compared to the reference category, while a HR less than 1 suggests a decreased risk. The dashed vertical line at HR = 1 represents no difference in risk compared to the reference. Significance levels are indicated by the p-values, with a p-value of less than 0.05 considered statistically significant.

Across the studied period, there were 201 all-cause deaths and 57 CVD deaths in our study cohort during the follow-up. We designed 3 Cox regression models to investigate the independent role of BMI levels in all-cause and CVD mortality. The adjusted HRs for all-cause and CVD mortality in relation to BMI categories are presented in Table 2 and visualized in the accompanying forest plot (Fig 2). The reference category for these models is the BMI range from 25.0 to 27.5 kg/m^2^, against which other categories are compared. The results indicate that after adjusting age, sex, race, arthritis, smoking status and heavy alcohol were added (Model 3), individuals with a BMI of less than 18.5 have a significantly higher risk of all-cause mortality (HR 5.31, 95% CI [2.08, 13.55], p < 0.001) when compared to the reference category. This elevated risk is similarly observed in the higher BMI categories for CVD mortality, with the HR for individuals with a BMI between 18.5 and 20.5 being 9.57 (95% CI [2.07, 44.26], p = 0.004), suggesting a substantially increased risk as well. Besides, the forest plot graphically represents these relationships in Fig 2. In summary, this analysis highlights a non-linear relationship between BMI and mortality, with both low and high BMI categories associated with increased risks of all-cause and CVD mortality, underscoring the complex interplay between BMI and health outcomes.

**Table 2:** HRs (95% CIs) for all-cause mortality according to BMI among participants with AFF.

BMI	Hazard ratio (95% CI) P value				
	Number of deaths (%)	Person years	Model 1	Model 2	Model 3
**All-cause mortality**					
<18.5	6 (42.86%)	1865	3.68 (1.52, 8.91) 0.004	4.28 (1.76, 10.44) 0.001	5.31 (2.08, 13.55) <0.001
18.5–20.5	8 (20.00%)	6767	1.26 (0.57, 2.78) 0.564	2.60 (1.16, 5.83) 0.020	4.31 (1.56, 11.89) 0.005
20.5–22.5	19 (25.33%)	12197	1.67 (0.93, 3.01) 0.086	1.82 (1.00, 3.29) 0.049	2.17 (1.05, 4.48) 0.036
22.5–25.0	40 (23.81%)	27807	1.54 (0.95, 2.51) 0.083	1.59 (0.97, 2.59) 0.064	1.54 (0.87, 2.75) 0.142
**25.0–27.5**	**27 (14.67%)**	**29796**	**1.00 (reference)**	**1.00 (reference)**	**1.00 (reference)**
27.5–30.0	35 (19.34%)	27960	1.39 (0.84, 2.30) 0.197	1.20 (0.72, 1.98) 0.483	1.21 (0.67, 2.19) 0.528
30.0–35.0	43 (19.37%)	36059	1.29 (0.80, 2.09) 0.296	1.07 (0.66, 1.74) 0.778	1.14 (0.65, 2.00) 0.636
≥ 35.0	23 (19.01%)	18406	1.39 (0.80, 2.43) 0.244	1.22 (0.69, 2.17) 0.489	1.39 (0.72, 2.70) 0.327
**CVD mortality**					
<18.5	1 (11.11%)	1446	2.42 (0.30, 19.34) 0.405	2.33 (0.29, 18.79) 0.426	3.72 (0.41, 33.74) 0.242
18.5–20.5	4 (11.11%)	6380	2.11 (0.64, 7.01) 0.223	3.36 (0.96, 11.81) 0.059	9.57 (2.07, 44.26) 0.004
20.5–22.5	6 (9.68%)	10818	1.88 (0.65, 5.43) 0.241	2.39 (0.82, 6.93) 0.110	3.10 (0.72, 13.31) 0.127
22.5–25.0	12 (10.00%)	24811	1.66 (0.68, 4.05) 0.269	1.73 (0.70, 4.25) 0.234	1.79 (0.57, 5.59) 0.318
**25.0–27.5**	**8 (4.85%)**	**27578**	**1.00 (reference)**	**1.00 (reference)**	**1.00 (reference)**
27.5–30.0	9 (5.81%)	25008	1.23 (0.48, 3.20) 0.665	1.10 (0.42, 2.85) 0.850	1.08 (0.31, 3.81) 0.901
30.0–35.0	9 (4.79%)	31842	0.98 (0.38, 2.54) 0.969	0.83 (0.32, 2.17) 0.709	0.91 (0.27, 3.04) 0.879
≥ 35.0	8 (7.55%)	16721	1.68 (0.63, 4.48) 0.298	1.31 (0.48, 3.62) 0.599	1.58 (0.44, 5.71) 0.485

Model 1: Non-adjusted; Model 2: Adjusted for age, sex, race; Model 3: Adjusted for age, sex, race, arthritis, smoking status and heavy alcohol.

### The detection of nonlinear relationships

**Fig 3:**
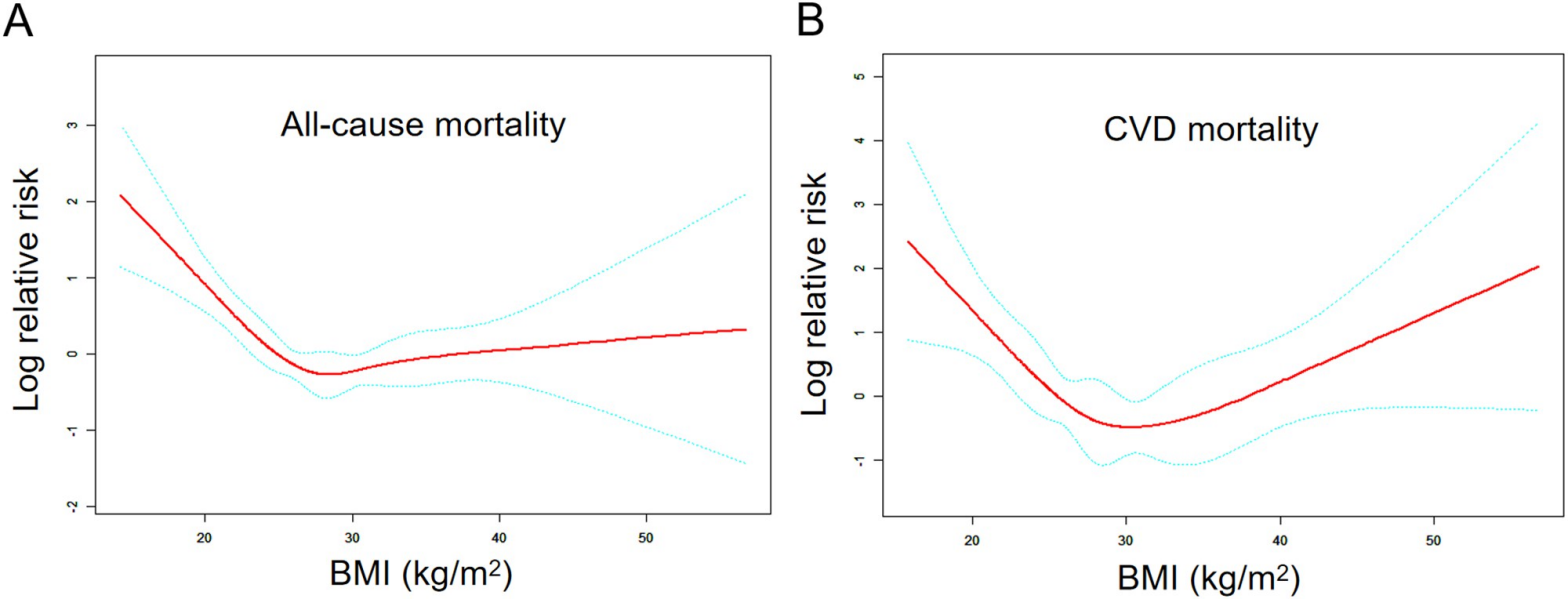
Associations of BMI with all-cause (A) and CVD (B) mortality in participants with AFF. Adjusted for age, sex, race, arthritis, smoking status and heavy alcohol. The red and blue lines represent the estimated values and their corresponding 95% CIs, respectively.

Using generalized additive models and smoothed curve fitting, we observed L-shaped and U-shaped relationships between BMI and all-cause and CVD mortality, respectively (Fig 3). Next, we employed a combination of a Cox proportional hazards model and a two-piecewise Cox proportional hazards model to explore the non-linear connection between BMI and mortality in AFF individuals (with a p-value for log-likelihood ratio <0.001) (Table 3). Our findings revealed that the inflection points for all-cause and CVD mortality occurred at 26.69 and 27.40 kg/m^2^. For BMI values below these inflection points (26.69 and 27.40 kg/m^2^), we observed a decrease in both all-cause and CVD mortality rates as BMI increased. The hazard ratios (HR) for all-cause and CVD mortality in this range were 0.83 (95% CI: 0.77, 0.89) and 0.76 (95% CI: 0.67, 0.87), respectively. However, when BMI exceeded these inflection points, we found no discernible association with all-cause mortality (HR: 1.03; 95% CI: 0.99, 1.07). In contrast, CVD mortality rates increased with higher BMI values beyond the inflection point (HR: 1.08; 95% CI: 1.00, 1.16).

**Table 3:** Threshold effect analysis of BMI on all-cause and CVD mortality in AFF individuals.

	Adjusted HR (95% CI) P-value
**All-cause mortality**	
Fitting by the two-piecewise linear model	
Inflection point	26.69
BMI (each 1 kg/m^2^ of BMI increase) < Inflection point	0.83 (0.77, 0.89) <0.001
BMI (each 1 kg/m^2^ of BMI increase) ≥ Inflection point	1.03 (0.99, 1.08) 0.136
P for Log-likelihood ratio	<0.001
**CVD mortality**	
Fitting by the two-piecewise linear model	
Inflection point	27.40
BMI (each 1 kg/m^2^ of BMI increase) < Inflection point	0.76 (0.67, 0.87) <0.001
BMI (each 1 kg/m^2^ of BMI increase) ≥ Inflection point	1.08 (1.00, 1.16) 0.045
P for Log-likelihood ratio	<0.001

Adjusted for age, sex, race, arthritis, smoking status and heavy alcohol.

### Stratified analyses

The stratified analysis demonstrated that a higher BMI (≥ 26.69 kg/m^2^) consistently offers a protective relationship compared to a lower BMI (< 26.69 kg/m^2^) for survival among individuals in AFF. Across most subgroups, higher BMI showed a generally favorable effect, further confirming the stability of our overall findings.

**Fig 4:**
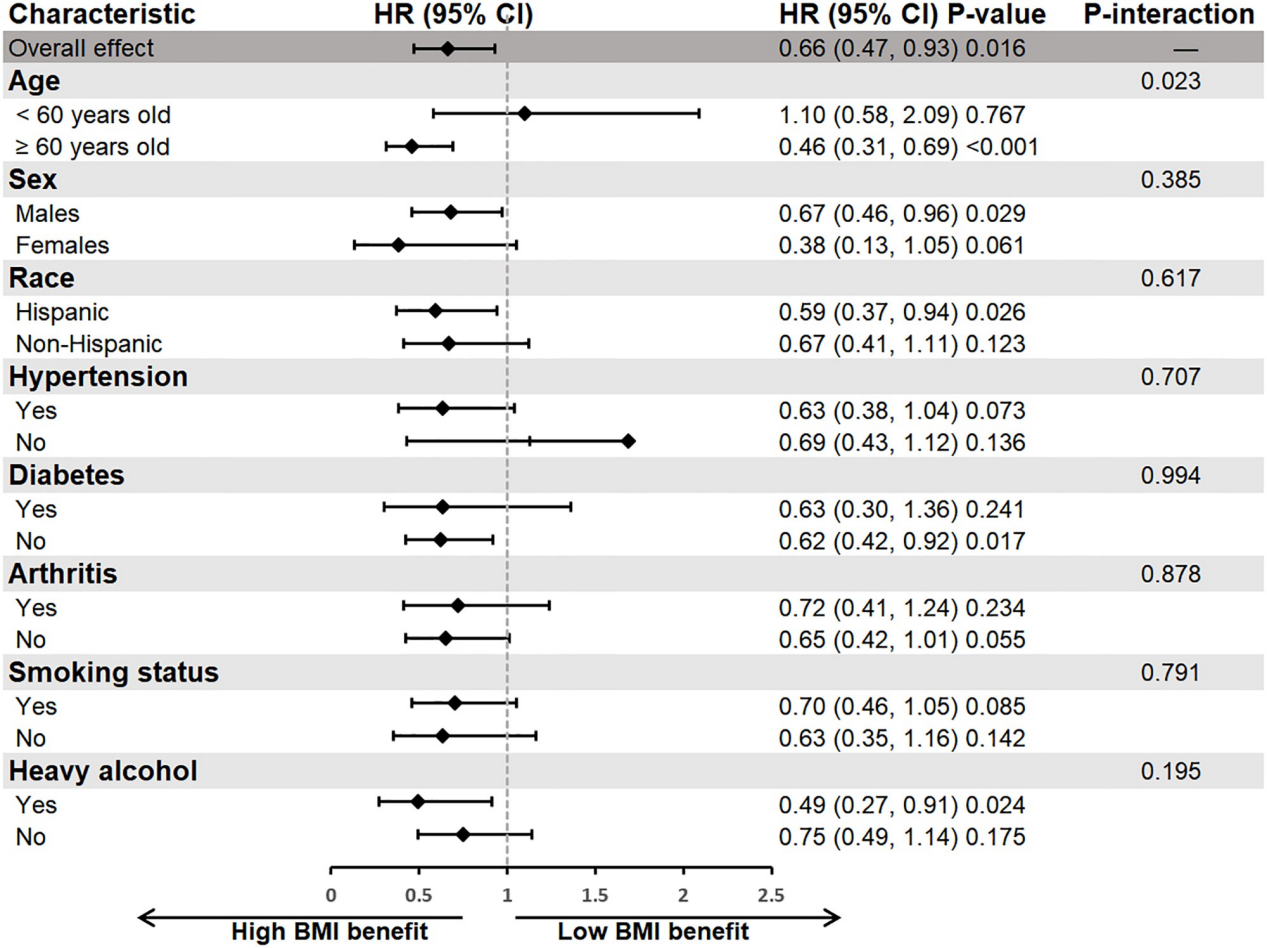
Forest plots of stratified analyses of BMI and all-cause mortality. Age, sex, race, arthritis, smoking status and heavy alcohol were all adjusted except the variable itself.

From the age group results, significantly reduced mortality risks were associated with higher BMI in older adults aged ≥ 60 years (HR: 0.46, 95% CI: 0.31, 0.69; p < 0.001), while the trend was not statistically significant in the younger cohort under 60 years old (HR: 1.10, 95% CI: 0.58, 2.09). Notably, the interaction test for age subgroups indicated a statistical difference (P-interaction = 0.023), suggesting that the impact of BMI on mortality may vary between different age groups. Consequently, we conducted further curve fitting analysis by age group to explore how BMI affects mortality rates within different life stages. This analysis helps us understand how BMI influences mortality risk at various points in the lifespan more comprehensively (**S1 Fig and S1 Table in** S1 Appendix). In other subgroups such as sex, race, hypertension, diabetes, arthritis, smoking status, and heavy alcohol consumption, although no significant interactions were observed, higher BMI generally showed a potential reduction in mortality risk. This trend emphasizes the potential general protective effect of higher BMI in individuals within the AFF (Fig 4).

In summary, the results of the stratified analysis support our primary findings that higher BMI is associated with lower mortality risk in the AFF, and this association is consistent across various demographic and behavioral subgroups (except for age).

## Discussion

This prospective cohort study, utilizing data from NHANES, examined the relationship between BMI and all-cause mortality among individuals in Agriculture, Forestry, and Fishing (AFF) occupations. Our findings revealed a unique L-shaped association between BMI and mortality in this population, indicating that before BMI exceeded 26.69 kg/m^2^, all-cause mortality decreased as BMI increased; however, above this threshold, the relationship between all-cause mortality and BMI was not statistically significant. This finding is crucial as it contrasts with common trends observed in the general population, underscoring the distinctive impact of occupational factors on health within the AFF sector.

Previous studies have thoroughly explored the relationship between body mass index (BMI) and mortality, often observing J-shaped or U-shaped patterns across populations [24, 26, 27]. Krishnan Bhaskaran analysis of 36 million UK adults revealed a J-shaped correlation between BMI and overall mortality, as well as with most specific causes of death [26]. Conversely, Xiaomin Sun’s research on people with obesity indicated a non-linear (U-shaped) correlation between BMI and all-cause mortality [28]. Likewise, Wei Qin’s study on heart failure patients also confirmed this U-shaped association with BMI [29]. These findings suggest that both low and high BMI levels are associated with increased mortality rates, with the lowest risk occurring in mid-range BMI categories. However, the optimal BMI for minimal mortality risk varies among different study cohorts, indicating influences from demographic, lifestyle, and potentially occupational factors. A prospective study of a middle-aged industrial cohort revealed that increased BMI is associated with higher mortality from various causes, such as cardiovascular diseases, diabetes, and accidents [30]. BMI and mortality rates showed several distinct differences between AFF workers and the general population. High physical activity in AFF occupations often led to greater muscle mass, resulting in higher BMIs but without the health risks typically seen in less active populations [31]. Our finding challenges the traditional view of BMI as a direct health risk indicator. Additionally, occupational hazards and lifestyle factors in these sectors altered the BMI-health outcome relationship [32]. In contrast to the general population, where higher BMI is linked to chronic diseases and increased mortality, AFF workers’ physically demanding work and distinct health profiles attenuate these risks [33, 34]. Socioeconomic factors, differing between AFF workers and the general population, influenced diet, lifestyle choices, and healthcare access. Often, AFF workers in remote or rural areas had limited healthcare access, affecting the management of BMI-related health issues [35]. The ’obesity paradox’, where higher BMI does not strongly correlate with increased mortality risk as in less active populations, may apply more to AFF workers [36]. Our study highlights the necessity for tailored health guidelines and interventions for specific occupations.

The association between BMI and mortality is complex, influenced by a range of physiological and metabolic processes [37]. Elevated BMI is frequently associated with a greater risk of chronic diseases such as type 2 diabetes, cardiovascular diseases, and certain cancers, which in turn raise mortality risks [38, 39]. Elevated BMI is often indicative of increased adiposity, which can lead to systemic inflammation and metabolic disturbances, such as insulin resistance—a key factor in the development of type 2 diabetes [40, 41]. Obesity compromises insulin efficiency in the body, resulting in higher blood sugar levels [42]. Furthermore, higher BMI values, indicative of obesity, are established risk factors for hypertension. Excess body fat strains the heart and impacts arteries, contributing to higher blood pressure [43]. Diabetes and hypertension significantly contribute to global mortality as key risk factors for cardiovascular diseases [44]. The relationship between BMI and mortality is multifaceted; higher BMI is linked to increased mortality risks like diabetes and cardiovascular diseases, whereas very low BMI may lead to heightened mortality risks, likely due to malnutrition and related health issues. This adiposity elevates the risk of cardiovascular diseases by promoting dyslipidemia and atherosclerosis [45]. Conversely, higher BMI may also indicate greater muscle mass and physical reserves, providing benefits in particular demographics, notably individuals in physically demanding occupations [46]. Increased muscle mass leading to a high BMI, especially in physically demanding occupations, is associated with enhanced insulin sensitivity and improved metabolic health [47]. A BMI between 18.5 and 25 kg/m^2^ is considered healthy, supporting essential physiological functions and disease resistance [48]. When BMI exceeds the normal range, reaching 25 to 26.69 kg/m^2^, increased body fat may offer protection against diseases like heart disease and certain cancers. This "obesity paradox" is prevalent among the elderly, possibly because additional body fat provides essential energy reserves during severe illness or surgery [49–51]. However, a BMI above 26.69 kg/m^2^ leads to excessive body fat, increasing risks of cardiovascular diseases, diabetes, respiratory issues, and some cancers [52]. These conditions are linked to chronic low-grade inflammation, with excessive body fat promoting inflammatory responses that accelerate chronic disease development [49]. Additionally, high BMI may cause metabolic disorders like insulin resistance, heightening diabetes risk [53]. Keeping BMI within an ideal range is essential for health. Although a slight increase in BMI might be beneficial in some cases, maintaining a healthy BMI is crucial to prevent various chronic diseases [54]. It is recommended to maintain weight through a balanced diet and regular exercise, avoiding extreme BMI values, to enhance long-term health and prevent diseases [55]. This comprehensive strategy aids in weight loss and enhances metabolic health.

The L-shaped BMI-mortality relationship observed in AFF workers suggests that while all-cause mortality decreases with increasing BMI up to a threshold of 26.69 kg/m^2^, beyond this point, the association between BMI and mortality does not show statistical significance. This finding contradicts the well-established notion that obesity, characterized by a high BMI, is a risk factor for increased mortality [56]. The unique characteristics of AFF occupations, including strenuous physical labor and environmental exposures, may contribute to this divergent relationship [20]. Several hypotheses can be proposed to explain this observed association. Firstly, the physically demanding nature of AFF jobs may require individuals to maintain a higher BMI to meet the energy demands of their work. A higher BMI could provide a metabolic advantage in coping with the strenuous and calorie-intensive tasks often encountered in these professions [57, 58]. Secondly, environmental exposures, such as exposure to agricultural pesticides or extreme weather conditions in fishing, may interact with BMI in complex ways [59]. It is possible that a higher BMI offers some protective effects against these environmental hazards, reducing mortality risk. Lastly, the age structure of the AFF workforce, with a significant proportion of older individuals, may also play a role in this relationship. Older workers may benefit from a higher BMI as they face age-related muscle loss and decreased physical capacity [60].

Our findings also indicate that AFF individuals have the lowest mortality rates at a BMI of 25–27.5 kg/m^2^, particularly at the critical inflection point of 26.69 kg/m^2^. This implies that mortality risk diminishes as BMI increases beyond the threshold. Several researches suggested that increased physical activity correlates with lower mortality rates, reinforcing the hypothesis that a higher BMI may be advantageous for the AFF workforce due to their rigorous physical exertion [61, 62]. The consistency of our findings across sex, race, hypertension, diabetes, arthritis, smoking status and heavy alcohol subgroups suggests that the L-shaped BMI-all cause mortality association remains robust across various demographic and behavioral factors within the AFF population. An inflection at a BMI of 26.69 kg/m^2^ signifies a critical threshold, marking a significant shift in associated mortality risk. This precise finding is particularly valuable for developing targeted health interventions and policies in the AFF sector. Epidemiologically, this inflection point challenges the traditional view of a linear or simple curvilinear BMI-all cause mortality relationship [63]. This discovery indicates a more complex, potentially occupation-specific relationship, showing significant BMI impact variations on health outcomes, influenced by occupational context and lifestyle factors. This insight is instrumental for public health practitioners and occupational health specialists. It enables the customization of BMI-related health recommendations to align better with the unique physical demands and risk profiles of AFF workers. Identifying occupation-specific health metrics is crucial for developing effective, evidence-based strategies to reduce occupational health disparities and enhance worker health. Additionally, this finding enriches the discussion on BMI as a universal health metric. It highlights the need for a nuanced approach in BMI assessment and interpretation, considering the interplay of occupational activities, lifestyle factors, and individual health risks. Therefore, the inflection point serves as a critical marker for AFF occupational health and a catalyst for re-evaluating BMI’s role in various epidemiological contexts.

### Strengths and limitations

Our study offers new insights into the BMI-all-cause mortality relationship within the Agriculture, Forestry, and Fishing (AFF) sector. The strengths of our study include a substantial sample size and the use of a well-regarded national database, which bolster the reliability of our findings. The interaction of occupational, environmental, and lifestyle factors renders the BMI-mortality correlation in the AFF sector particularly complex. The specific physical demands and risks associated with these professions necessitate a refined analysis of health indicators such as BMI. The identification of a specific BMI threshold (26.69 kg/m^2^) marking a change in mortality risk represents a valuable and precise contribution to health guidelines for AFF occupations. This study adds to the accumulating evidence that universal health guidelines may not be applicable universally, especially in professions characterized by unique physical and environmental demands. However, the study has limitations, including the use of self-reported occupational data, which may introduce misclassification bias. Although the study considers several covariates, unmeasured factors specific to AFF occupations may also influence the results. Being a cohort study, it identifies associations rather than establishing causality. Inherent biases in observational data might influence the findings. Furthermore, BMI’s inability to differentiate fat from muscle mass may cause body composition misclassification, a significant concern for physically active groups such as AFF workers [64]. Future research should investigate the physiological and occupational factors that contribute to this unique association. Further research should also assess the overall health impact of BMI, beyond mortality, in AFF occupations. In conclusion, this study provides valuable insights into the BMI-mortality relationship in a specific occupational sector, underscoring the need for tailored health guidelines. However, its specialized focus and observational nature limit the findings’ broader applicability, necessitating further research for a more comprehensive understanding. Meanwhile, within the AFF category, there are differences among different occupations in agriculture, forestry, and fishing, but the NHANES database records AFF as a single unified category, making it impossible to analyze the relationship between BMI and mortality separately for each occupation. These occupations exhibit varying degrees of health risk exposure, influenced by their specific work environments and physical demands. For example, agricultural workers are exposed to prolonged sunlight and pesticides, forestry workers handle complex terrain and heavy machinery, and fishing industry workers frequently encounter harsh weather and water hazards. This categorization neglects significant risk differences among these professions, potentially affecting the accurate understanding and assessment of occupational health effects. Consequently, future research should acknowledge the distinctiveness of these occupations, conducting more detailed subdivisions and analyses to better understand how different work environments affect health. This analysis would not only yield more precise data but also assist in developing targeted occupational health measures to mitigate health risks for specific worker groups.

## Conclusions

This prospective cohort study, utilizing NHANES data from 1999 to 2014, revealed a unique L-shaped association between Body Mass Index (BMI) and all-cause mortality in individuals engaged in Agriculture, Forestry, and Fishing (AFF) occupations. All-cause mortality declined with increasing BMI up to 26.69 kg/m^2^, but beyond this threshold, the relationship was not statistically significant. This novel finding contradicts general population trends and suggests that individuals in AFF occupations may benefit from a certain range of higher BMI in terms of reduced mortality risk. The physically demanding nature of AFF jobs and exposure to environmental hazards specific to these professions may contribute to this unique BMI-mortality relationship. Further research is warranted to explore the underlying mechanisms and elucidate the role of BMI as a health indicator in this occupational group. Tailored health guidelines and risk assessments should be considered for individuals in AFF occupations, acknowledging their distinct health needs and the potential advantages of a higher BMI in this context.

## Supporting information

S1 Appendix Contains S1 Fig and S1 Table. (DOC)

## References

[1] Wolfenden L, Ezzati M, Larijani B, Dietz W. The challenge for global health systems in preventing and managing obesity. Obes Rev. 2019;20 Suppl 2:185–93. Epub 2019/07/19. doi: 10.1111/obr.12872 31317659

[2] Kompaniyets L, Freedman DS, Belay B, Pierce SL, Kraus EM, Blanck HM, et al. Probability of 5% or Greater Weight Loss or BMI Reduction to Healthy Weight Among Adults With Overweight or Obesity. JAMA Netw Open. 2023;6(8):e2327358. Epub 2023/08/07. doi: 10.1001/jamanetworkopen.2023.27358 37548978 PMC10407685

[3] WHO. Physical Status: The Use and Interpretation of Anthropometry: Report of a World Health Organization. 1995/01/01 ed. Geneva, Switzerland World Health Organization; 1995. 1–452 p. 8594834

[4] Khan I, Chong M, Le A, Mohammadi-Shemirani P, Morton R, Brinza C, et al. Surrogate Adiposity Markers and Mortality. JAMA Netw Open. 2023;6(9):e2334836. Epub 2023/09/20. doi: 10.1001/jamanetworkopen.2023.34836 37728925 PMC10512100

[5] Zhao W, Katzmarzyk PT, Horswell R, Wang Y, Li W, Johnson J, et al. Body mass index and the risk of all-cause mortality among patients with type 2 diabetes mellitus. Circulation. 2014;130(24):2143–51. Epub 2014/11/08. doi: 10.1161/CIRCULATIONAHA.114.009098 25378546 PMC4302029

[6] Ruder K. Mounting Evidence Suggests That BMI Isn’t the Only Measure Needed to Predict Mortality Risk. Jama. 2023;330(6):490–1. Epub 2023/07/19. doi: 10.1001/jama.2023.13602 37466937

[7] Gu JK, Charles LE, Fekedulegn D, Ma CC, Andrew ME, Burchfiel CM. Prevalence of Injury in Occupation and Industry: Role of Obesity in the National Health Interview Survey 2004 to 2013. J Occup Environ Med. 2016;58(4):335–43. doi: 10.1097/JOM.0000000000000670 27058472 PMC4922363

[8] Wada K, Eguchi H, Prieto-Merino D. Differences in stroke and ischemic heart disease mortality by occupation and industry among Japanese working-aged men. SSM-population health. 2016;2:745–9. doi: 10.1016/j.ssmph.2016.10.004 29349185 PMC5757844

[9] Zhang B, Dong X. The unique association between serum 25-hydroxyvitamin D concentrations and blood lipid profiles in agriculture, forestry, and fishing occupations: Insights from NHANES 2001–2014. PLoS One. 2024;19(2):e0297873. doi: 10.1371/journal.pone.0297873 38412162 PMC10898752

[10] Fan ZJ, Bonauto DK, Foley MP, Silverstein BA. Underreporting of work-related injury or illness to workers’ compensation: individual and industry factors. Journal of Occupational and Environmental Medicine. 2006;48(9):914–22. doi: 10.1097/01.jom.0000226253.54138.1e 16966958

[11] Zhao G, Ronda E, Cea L, Pulido J, Barrio G, Regidor E. Mortality by cause of death and risk behaviors in farmers versus non-farmers: the importance of avoiding the healthy worker effect. International Archives of Occupational and Environmental Health. 2019;92:599–608. doi: 10.1007/s00420-018-1396-2 30603873

[12] Lee H-E, Kim H-R, Chung YK, Kang S-K, Kim E-A. Mortality rates by occupation in Korea: a nationwide, 13-year follow-up study. Occup Environ Med. 2016;73(5):329–35. doi: 10.1136/oemed-2015-103192 26920855 PMC4853594

[13] Abdullah A, Wolfe R, Stoelwinder JU, de Courten M, Stevenson C, Walls HL, et al. The number of years lived with obesity and the risk of all-cause and cause-specific mortality. Int J Epidemiol. 2011;40(4):985–96. Epub 2011/03/02. doi: 10.1093/ije/dyr018 21357186

[14] Kuk JL, Ardern CI. Are metabolically normal but obese individuals at lower risk for all-cause mortality? Diabetes Care. 2009;32(12):2297–9. Epub 2009/09/05. doi: 10.2337/dc09-0574 19729521 PMC2782994

[15] Avgerinos KI, Spyrou N, Mantzoros CS, Dalamaga M. Obesity and cancer risk: Emerging biological mechanisms and perspectives. Metabolism. 2019;92:121–35. Epub 2018/11/18. doi: 10.1016/j.metabol.2018.11.001 30445141

[16] Fontana L, Hu FB. Optimal body weight for health and longevity: bridging basic, clinical, and population research. Aging Cell. 2014;13(3):391–400. Epub 2014/03/19. doi: 10.1111/acel.12207 24628815 PMC4032609

[17] De Mutsert R, Sun Q, Willett WC, Hu FB, van Dam RM. Overweight in early adulthood, adult weight change, and risk of type 2 diabetes, cardiovascular diseases, and certain cancers in men: a cohort study. Am J Epidemiol. 2014;179(11):1353–65. Epub 2014/05/03. doi: 10.1093/aje/kwu052 24786797 PMC4036209

[18] De Bandt JP, Monin C. Obesity, Nutrients and the Immune System in the Era of COVID-19. Nutrients. 2021;13(2). Epub 2021/03/07. doi: 10.3390/nu13020610 33668493 PMC7917599

[19] Chapman LJ, Husberg B. Agriculture, forestry, and fishing sector. J Safety Res. 2008;39(2):171–3. Epub 2008/05/06. doi: 10.1016/j.jsr.2008.02.008 18454963

[20] Quandt SA, Kucera KL, Haynes C, Klein BG, Langley R, Agnew M, et al. Occupational health outcomes for workers in the agriculture, forestry and fishing sector: implications for immigrant workers in the southeastern US. Am J Ind Med. 2013;56(8):940–59. Epub 2013/03/02. doi: 10.1002/ajim.22170 23450720

[21] Scott E, Hirabayashi L, Luschen K, Krupa N, Jenkins P. Ensuring data quality and maximizing efficiency in coding agricultural and forestry injuries: Lessons to improve occupational injury surveillance. J Safety Res. 2022;83:323–8. Epub 2022/12/09. doi: 10.1016/j.jsr.2022.09.006 36481023

[22] Damalas CA, Koutroubas SD. Farmers’ Exposure to Pesticides: Toxicity Types and Ways of Prevention. Toxics. 2016;4(1). Epub 2016/01/08. doi: 10.3390/toxics4010001 29051407 PMC5606636

[23] Scott E, Hirabayashi L, Graham J, Krupa N, Jenkins P. Using hospitalization data for injury surveillance in agriculture, forestry and fishing: a crosswalk between ICD10CM external cause of injury coding and The Occupational Injury and Illness Classification System. Inj Epidemiol. 2021;8(1):6. Epub 2021/02/16. doi: 10.1186/s40621-021-00300-6 33583430 PMC7883573

[24] Lee DH, Keum N, Hu FB, Orav EJ, Rimm EB, Willett WC, et al. Predicted lean body mass, fat mass, and all cause and cause specific mortality in men: prospective US cohort study. Bmj. 2018;362:k2575. Epub 2018/07/05. doi: 10.1136/bmj.k2575 29970408 PMC6028901

[25] NHANES, Tutorials. https://wwwn.cdc.gov/nchs/nhanes/tutorials/default.aspx 2022 [Accessed March 20 2022].

[26] Bhaskaran K, Dos-Santos-Silva I, Leon DA, Douglas IJ, Smeeth L. Association of BMI with overall and cause-specific mortality: a population-based cohort study of 3·6 million adults in the UK. Lancet Diabetes Endocrinol. 2018;6(12):944–53. Epub 2018/11/06. doi: 10.1016/s2213-8587(18)30288-2 30389323 PMC6249991

[27] Aune D, Sen A, Prasad M, Norat T, Janszky I, Tonstad S, et al. BMI and all cause mortality: systematic review and non-linear dose-response meta-analysis of 230 cohort studies with 3.74 million deaths among 30.3 million participants. Bmj. 2016;353:i2156. Epub 2016/05/06. doi: 10.1136/bmj.i2156 27146380 PMC4856854

[28] Sun X, Yan AF, Shi Z, Zhao B, Yan N, Li K, et al. Health consequences of obesity and projected future obesity health burden in China. Obesity (Silver Spring). 2022;30(9):1724–51. Epub 2022/08/25. doi: 10.1002/oby.23472 36000246

[29] Qin W, Liu F, Wan C. A U-shaped association of body mass index and all-cause mortality in heart failure patients: A dose-response meta-analysis of prospective cohort studies. Cardiovasc Ther. 2017;35(2). Epub 2016/10/27. doi: 10.1111/1755-5922.12232 27783461

[30] Tsai SP, Donnelly RP, Wendt JK. Obesity and mortality in a prospective study of a middle-aged industrial population. J Occup Environ Med. 2006;48(1):22–7. Epub 2006/01/13. doi: 10.1097/01.jom.0000184866.49000.e5 16404206

[31] Pickett W, King N, Lawson J, Dosman JA, Trask C, Brison RJ, et al. Farmers, mechanized work, and links to obesity. Prev Med. 2015;70:59–63. Epub 2014/12/03. doi: 10.1016/j.ypmed.2014.11.012 25448840

[32] Schulte PA, Wagner GR, Ostry A, Blanciforti LA, Cutlip RG, Krajnak KM, et al. Work, obesity, and occupational safety and health. Am J Public Health. 2007;97(3):428–36. Epub 2007/02/03. doi: 10.2105/AJPH.2006.086900 17267711 PMC1805035

[33] Larsson SC, Burgess S. Causal role of high body mass index in multiple chronic diseases: a systematic review and meta-analysis of Mendelian randomization studies. BMC Med. 2021;19(1):320. Epub 2021/12/16. doi: 10.1186/s12916-021-02188-x 34906131 PMC8672504

[34] Butler-Laporte G, Harroud A, Forgetta V, Richards JB. Elevated body mass index is associated with an increased risk of infectious disease admissions and mortality: a mendelian randomization study. Clin Microbiol Infect. 2020. Epub 2020/06/28. doi: 10.1016/j.cmi.2020.06.014 32592749

[35] Frank AL, Liebman AK, Ryder B, Weir M, Arcury TA. Health care access and health care workforce for immigrant workers in the agriculture, forestry, and fisheries sector in the southeastern US. Am J Ind Med. 2013;56(8):960–74. Epub 2013/03/28. doi: 10.1002/ajim.22183 23532981

[36] McAuley PA, Blair SN. Obesity paradoxes. J Sports Sci. 2011;29(8):773–82. Epub 2011/03/19. doi: 10.1080/02640414.2011.553965 21416445

[37] Li J, Simon G, Castro MR, Kumar V, Steinbach MS, Caraballo PJ. Association of BMI, comorbidities and all-cause mortality by using a baseline mortality risk model. PLoS One. 2021;16(7):e0253696. Epub 2021/07/10. doi: 10.1371/journal.pone.0253696 34242241 PMC8270162

[38] Silveira EA, da Silva Filho RR, Spexoto MCB, Haghighatdoost F, Sarrafzadegan N, de Oliveira C. The Role of Sarcopenic Obesity in Cancer and Cardiovascular Disease: A Synthesis of the Evidence on Pathophysiological Aspects and Clinical Implications. Int J Mol Sci. 2021;22(9). Epub 2021/05/01. doi: 10.3390/ijms22094339 33919368 PMC8122649

[39] Simati S, Kokkinos A, Dalamaga M, Argyrakopoulou G. Obesity Paradox: Fact or Fiction? Curr Obes Rep. 2023;12(2):75–85. Epub 2023/02/23. doi: 10.1007/s13679-023-00497-1 36808566

[40] Zatterale F, Longo M, Naderi J, Raciti GA, Desiderio A, Miele C, et al. Chronic Adipose Tissue Inflammation Linking Obesity to Insulin Resistance and Type 2 Diabetes. Front Physiol. 2019;10:1607. Epub 2020/02/18. doi: 10.3389/fphys.2019.01607 32063863 PMC7000657

[41] Wu H, Ballantyne CM. Metabolic inflammation and insulin resistance in obesity. Circulation research. 2020;126(11):1549–64. doi: 10.1161/CIRCRESAHA.119.315896 32437299 PMC7250139

[42] Abranches MV, Oliveira FC, Conceição LL, Peluzio MD. Obesity and diabetes: the link between adipose tissue dysfunction and glucose homeostasis. Nutr Res Rev. 2015;28(2):121–32. Epub 2015/12/10. doi: 10.1017/S0954422415000098 26650242

[43] Brown CD, Higgins M, Donato KA, Rohde FC, Garrison R, Obarzanek E, et al. Body mass index and the prevalence of hypertension and dyslipidemia. Obes Res. 2000;8(9):605–19. Epub 2001/02/28. doi: 10.1038/oby.2000.79 11225709

[44] Bloomfield GS, Wang TY, Boulware LE, Califf RM, Hernandez AF, Velazquez EJ, et al. Implementation of management strategies for diabetes and hypertension: from local to global health in cardiovascular diseases. Glob Heart. 2015;10(1):31–8. Epub 2015/03/11. doi: 10.1016/j.gheart.2014.12.010 25754564 PMC4754665

[45] Khafagy R, Dash S. Obesity and Cardiovascular Disease: The Emerging Role of Inflammation. Front Cardiovasc Med. 2021;8:768119. Epub 2021/11/12. doi: 10.3389/fcvm.2021.768119 34760952 PMC8573144

[46] Sergi TE, Bode KB, Hildebrand DA, Dawes JJ, Joyce JM. Relationship between Body Mass Index and Health and Occupational Performance among Law Enforcement Officers, Firefighters, and Military Personnel: A Systematic Review. Curr Dev Nutr. 2023;7(1):100020. Epub 2023/05/14. doi: 10.1016/j.cdnut.2022.100020 37181120 PMC10100923

[47] Wali JA, Solon-Biet SM, Freire T, Brandon AE. Macronutrient Determinants of Obesity, Insulin Resistance and Metabolic Health. Biology (Basel). 2021;10(4). Epub 2021/05/01. doi: 10.3390/biology10040336 33923531 PMC8072595

[48] Di Angelantonio E, Bhupathiraju SN, Wormser D, Gao P, Kaptoge S, De Gonzalez AB, et al. Body-mass index and all-cause mortality: individual-participant-data meta-analysis of 239 prospective studies in four continents. The Lancet. 2016;388(10046):776–86. doi: 10.1016/S0140-6736(16)30175-1 27423262 PMC4995441

[49] Flegal KM, Kit BK, Orpana H, Graubard BI. Association of all-cause mortality with overweight and obesity using standard body mass index categories: a systematic review and meta-analysis. Jama. 2013;309(1):71–82. doi: 10.1001/jama.2012.113905 23280227 PMC4855514

[50] Oreopoulos A, Kalantar-Zadeh K, Sharma AM, Fonarow GC. The obesity paradox in the elderly: potential mechanisms and clinical implications. Clinics in geriatric medicine. 2009;25(4):643–59. doi: 10.1016/j.cger.2009.07.005 19944265

[51] Casas-Vara A, Santolaria F, Fernández-Bereciartúa A, González-Reimers E, García-Ochoa A, Martínez-Riera A. The obesity paradox in elderly patients with heart failure: analysis of nutritional status. Nutrition. 2012;28(6):616–22. doi: 10.1016/j.nut.2011.10.006 22261572

[52] Caspersen CJ, Pereira MA, Curran KM. Changes in physical activity patterns in the United States, by sex and cross-sectional age. Med Sci Sports Exercise. 2000;32(9):1601–9. doi: 10.1097/00005768-200009000-00013 10994912

[53] Patel AV, Hildebrand JS, Gapstur SM. Body mass index and all-cause mortality in a large prospective cohort of white and black US adults. PLoS One. 2014;9(10):e109153. 25295620 10.1371/journal.pone.0109153 PMC4189918

[54] Nuttall FQ. Body mass index: obesity, BMI, and health: a critical review. Nutrition today. 2015;50(3):117–28. doi: 10.1097/NT.0000000000000092 27340299 PMC4890841

[55] Udani JK, Singh BB, Singh VJ, Barrett ML. Effects of Açai (Euterpe oleracea Mart.) berry preparation on metabolic parameters in a healthy overweight population: A pilot study. Nutr J. 2011;10:1–7. 21569436 10.1186/1475-2891-10-45 PMC3118329

[56] Shaukat A, Dostal A, Menk J, Church TR. BMI Is a Risk Factor for Colorectal Cancer Mortality. Dig Dis Sci. 2017;62(9):2511–7. Epub 2017/07/25. doi: 10.1007/s10620-017-4682-z 28733869

[57] Fujishiro K, Lawson CC, Hibert EL, Chavarro JE, Rich-Edwards JW. Job strain and changes in the body mass index among working women: a prospective study. Int J Obes (Lond). 2015;39(9):1395–400. Epub 2015/05/20. doi: 10.1038/ijo.2015.91 25986779 PMC4564350

[58] Bixby H, Bentham J, Zhou B, Di Cesare M, Paciorek CJ, Bennett JE, et al. Rising rural body-mass index is the main driver of the global obesity epidemic in adults. Nature. 2019;569(7755):260–4. doi: 10.1038/s41586-019-1171-x 31068725 PMC6784868

[59] Arcury TA, Grzywacz JG, Sidebottom J, Wiggins MF. Overview of immigrant worker occupational health and safety for the agriculture, forestry, and fishing (AgFF) sector in the southeastern United States. Am J Ind Med. 2013;56(8):911–24. Epub 2013/03/02. doi: 10.1002/ajim.22173 23450742

[60] Aiken-Morgan AT, Capuano AW, Wilson RS, Barnes LL. Changes in Body Mass Index and Incident Mild Cognitive Impairment Among African American Older Adults. J Gerontol A Biol Sci Med Sci. 2023. Epub 2023/11/14. doi: 10.1093/gerona/glad263 37962543 PMC10876072

[61] Suran M. Study: Short Spurts of Vigorous Physical Activity During Daily Life Are Associated With Lower Mortality. Jama. 2023;329(4):275–6. Epub 2023/01/05. doi: 10.1001/jama.2022.24054 36598767

[62] Kim D, Murag S, Cholankeril G, Cheung A, Harrison SA, Younossi ZM, et al. Physical Activity, Measured Objectively, Is Associated With Lower Mortality in Patients With Nonalcoholic Fatty Liver Disease. Clin Gastroenterol Hepatol. 2021;19(6):1240–7.e5. Epub 2020/07/20. doi: 10.1016/j.cgh.2020.07.023 32683103

[63] Bradbury KE, Cairns BJ. Understanding the relation between BMI and mortality. Bmj. 2019;364:l1219. Epub 2019/04/09. doi: 10.1136/bmj.l1219 30957772

[64] Zhu P, Li A, Cai Q, Chen Y, Liu Y, Jager-Wittenaar H, et al. Sex differences in the association between dual-energy x-ray absorptiometry-measured body composition and periodontitis. J Periodontol. 2023. Epub 2023/07/28. doi: 10.1002/JPER.23-0162 37505475

