# Supplementary material for: Associations between body mass index and all-cause and CVD mortality in agriculture, forestry, and fishing occupations: A prospective cohort study using NHANES data (1999–2014)
Source: PLoS One. 2024 Jul 8;19(7):e0305922. doi: 10.1371/journal.pone.0305922 (PMC11230546; doi:10.1371/journal.pone.0305922)
Supplement: S1 Appendix — (DOC) [file pone.0305922.s001.doc]

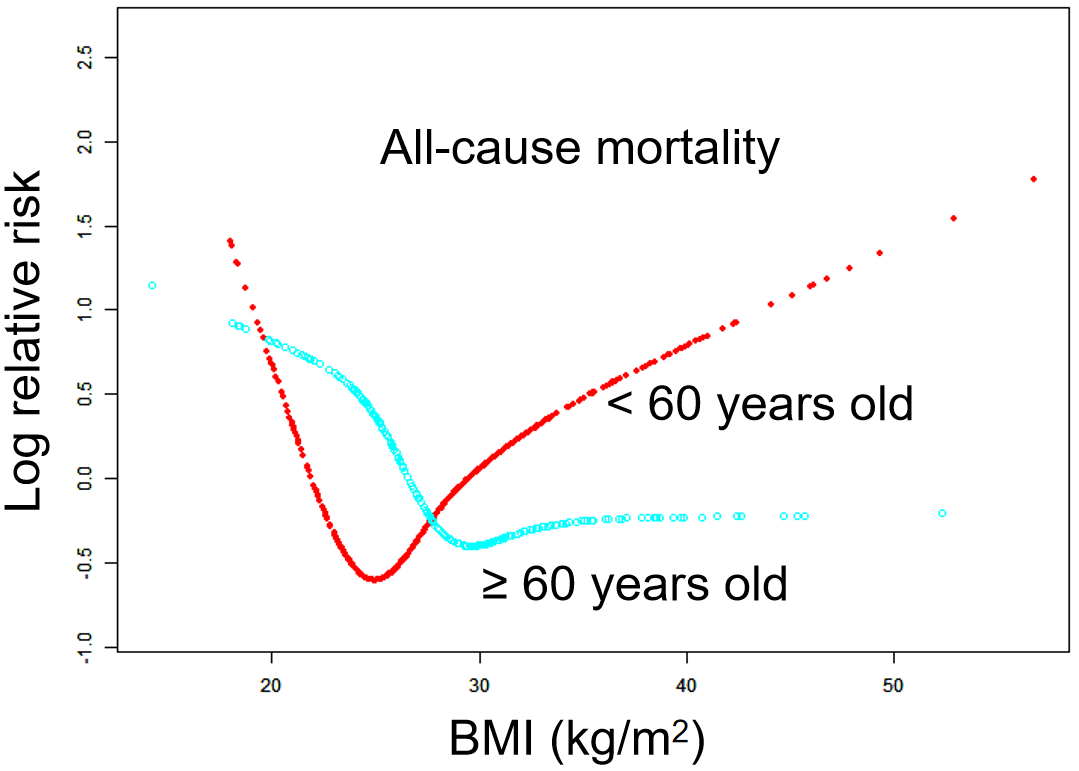


**Supplementary figure 1**. **Correlation Between BMI and All-Cause Mortality in participants with AFF** **stratified by age.** Adjusted for sex, race, arthritis, smoking status and heavy alcohol. The red line indicates the association in participants younger than 60 years, demonstrating a U-shaped curve. The blue line represents participants 60 years and older, illustrating an L-shaped curve.

**Supplementary table 1. Threshold effect analysis of BMI on all-cause mortality in AFF individuals stratified by age.**

|  | **Adjusted HR (95% CI) P-value** |
| --- | --- |
| **< 60 years old** |  |
| Fitting by the two-piecewise linear model |  |
| Infection point | 21.27 |
| BMI < Infection point | 0.55 (0.42, 0.74) <0.001 |
| BMI ≥ Infection point | 1.07 (1.01, 1.13) 0.017 |
| P for Log-likelihood ratio | <0.001 |
| **≥ 60 years old** |  |
| Fitting by the two-piecewise linear model |  |
| Infection point | 30.19 |
| BMI < Infection point | 0.89 (0.83, 0.94) <0.001 |
| BMI ≥ Infection point | 1.01 (0.94, 1.09) 0.721 |
| P for Log-likelihood ratio | 0.029 |

Adjusted for sex, race, arthritis, smoking status and heavy alcohol.
